# Development of Chemiluminescent Lateral Flow Assay for the Detection of Nucleic Acids

**DOI:** 10.3390/bios2010032

**Published:** 2012-01-18

**Authors:** Yuhong Wang, Catherine Fill, Sam R. Nugen

**Affiliations:** Department of Food Science, University of Massachusetts, 102 Holdsworth Way, Amherst, MA 01003, USA; E-Mails: yuhongw@foodsci.umass.edu (Y.W.); cfill@foodsci.umass.edu (C.F.)

**Keywords:** biosensor, lateral flow assay, *Trypanosoma*, chemiluminescence

## Abstract

Rapid, sensitive detection methods are of utmost importance for the identification of pathogens related to health and safety. Herein we report the development of a nucleic acid sequence-based lateral flow assay which achieves a low limit of detection using chemiluminescence. On-membrane enzymatic signal amplification is used to reduce the limit of detection to the sub-femtomol level. To demonstrate this assay, we detected synthetic nucleic acid sequences representative of *Trypanosoma* mRNA, the causative agent for African sleeping sickness, with relevance in human and animal health in sub-Saharan Africa. The intensity of the chemiluminescent signal was evaluated by using a charge-coupled device as well as a microtiter plate reader. We demonstrated that our lateral flow chemiluminescent assay has a very low limit of detection and is easy to use. The limit of detection was determined to be 0.5 fmols of nucleic acid target.

## 1. Introduction

*Trypanosoma* such as *Trypanosoma brucei* and *Trypanosoma cruzi*, the causative agents for the potentially fatal African sleeping sickness and Chagas disease, respectively, have important influence on human health. African sleeping sickness occurs mainly in sub-Saharan Africa countries where it was reported that about 10,000 people were infected in 2009 [1,2]. Chagas disease is found in the Americas, mainly in developing areas of Latin America [1]. The currently accepted methods for the detection of *Trypanosoma* such as microscopic examination and xenodiagnoses have poor sensitivity and are also labor-intensive and time-consuming [1,2]. Immunological methods such as enzyme-linked immunosorbent assay, immunochromatographic dipstick test, radioimmunosorbent assay, and immunofluorescence antibody test are rapid and sensitive but not specific [3,4,5,6,7,8,9]. Molecular methods such as PCR and real-time nucleic acid sequence-based amplification are very specific but expensive and time consuming, although combination of PCR and chemiluminescence southern blot has been used to improve the sensitivity of the detection of *Trypanosoma* [10,11,12,13,14,15,16,17]. These techniques are not implemented in Trypanosomiasis control programs due to the high cost of the equipment [14,16,18]. None of these methods are ideal to mass screening of samples such as the onset of outbreaks, epidemiological surveys, or blood unit screening [1], and without rapid and accurate diagnoses, treatment of the corresponding diseases is unlikely. Therefore, there is a need for an assay which can rapidly, sensitively and specifically detect *Trypanosoma* without the need for specialized equipment and highly trained personnel. 

Lateral flow assays are inexpensive and easy to use diagnostic methods which make them ideal for use in resource-limited areas such as those affected by *Trypanosoma* [19,20]. While gold nanoparticles are commonly used for lateral flow assays, other particles such as liposomes [21,22,23] have also been investigated to lower the limit of detection [19]. Chemiluminescence offers a unique method of signal amplification, in which horseradish peroxidase-labeled reporter probes catalyze luminol and hydrogen peroxide to generate a signal which can be quantified by chemiluminescent readers. The incorporation of chemiluminescence onto a lateral flow assay format has previously demonstrated improved sensitivity over colloidal gold [24]. Similarly, HRP amplification has also previously been used for chromogenic signal enhancement in a nucleic acid lateral flow assay [25]. These results demonstrate the potential of on-membrane enzymatic amplification for enhanced signal generation. In this study, a simple and sensitive chemiluminescent lateral flow assay using a horseradish peroxidase (HRP)-labeled reporter probe has been developed for nucleic acid-based detection of *Trypanosoma* mRNA sequences. The capture and reporter probes were designed to target the *Trypanosoma* mRNA leader sequence, a 35 nucleotide spliced sequence present on the 5' end of all *Trypanosoma* mRNA, and the poly A tail found on the 3' end, respectively [26,27]. We demonstrate the ability to detect sub-femtomol amounts of synthetic leader sequences representative of *Trypanosoma* mRNA. The resulting chemiluminescent lateral flow assay represents an inexpensive, rapid, and sensitive method for nucleic acid detection without the need for target amplification or costly equipment.

## 2. Experimental Section

**Figure 1:**
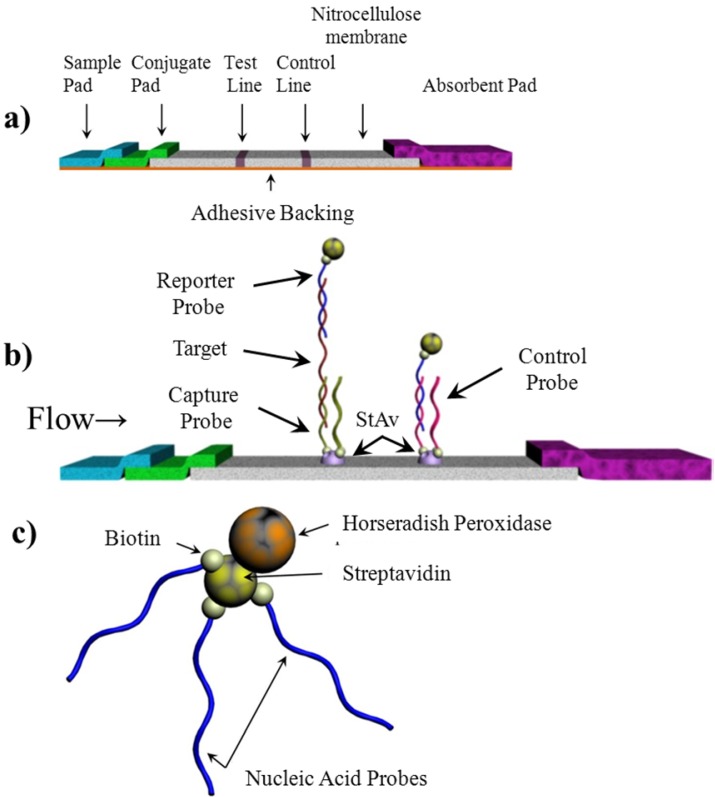
Schematic diagram of lateral flow test strip. (**a**) a typical structure of the lateral flow test strip; (**b**) Seen on the membrane are the “test line” and “control line”. The test line quantifies the target in the sample while the control line confirms adequate hybridization conditions. The capture and control probes are immobilized on the membrane prior to the assay. HRP indicates the horseradish peroxidase and StAv indicates streptavidin; (**c**) conjugation between HRP and nucleic acid via the interaction between biotin and streptavidin.

The test strip consists of four components mounted together on an adhesive backing: sample application pad, oligonucleotide-biotin-streptavidin-HRP conjugate release pad, nitrocellulose membrane, and the absorbent pad (Figure 1). Each component was prepared separately and then assembled on an adhesive backing prior to use. Once assembled, the strips were stored desiccated at 4 °C until use.

### 2.1. Capture and Reporter Probe Design

*Trypanosoma* mRNA was selected as the target analyte to demonstrate our chemiluminescent lateral flow assay, and synthetic nucleic acid sequences representing the mRNA were used in the experiments (see sequences in Table 1). The unique leader sequence at the 5' all *Trypanosoma* mRNA can serve as a unique probe hybridization site. In addition, the poly(A) tail on the 3' end can be found on all mRNA originating from eukaryotic organisms. These two regions of the mRNA were chosen as the probe binding sites. Both the reporter and capture probes were acquired with a biotin modification allowing for conjugation to streptavidin. The target sequence selected consisted of a leader sequence, a linker sequence and a 25 base poly(A) tail.

**Table 1:** Nucleic Acid Sequences used for the experiments.

Target Sequence	AAC GCT ATT ATT AGA ACA GTT TCT GTA CTA TAT TGA ACA TCA AGC AAA GAA AAT AAA TGC AGT TTT CAA AAA AAA AAA AAA AAA AAA AAA AA
Leader Sequence	AAC GCT ATT ATT AGA ACA GTT TCT GTA CTA TAT TG-biotin
Reporter Probe: Leader Sequence Complement	CAA TAT AGT ACA GAA ACT GTT CTA ATA ATA GCG TT-biotin
Capture Probe: Poly (A)_25_	biotin-AAA AAA AAA AAA AAA AAA AAA AAA A
Control Probe: Oligo d(T)_25_	biotin-TTT TTT TTT TTT TTT TTT TTT TTT T

### 2.2. Lateral Flow Assay Fabrication

#### 2.2.1. Sample Pad

The sample application pad (CF5, Whatman International Ltd., Piscataway, NJ) was pretreated by immersion in 0.1 M Na_2_B_4_O_7_·7H_2_O, pH 8.6 containing 1% Triton X-100 and was dried overnight at 37 °C. Following pretreatment, the pad was stored at room temperature in a desiccator until assembled. During the assay, the sample is placed on this pad and then migrates to the conjugate pad using capillary flow.

#### 2.2.2. Conjugate Pad

Streptavidin-tagged HRP (Thermo Fisher Scientific Inc., Rockford, IL) was bound to biotin-tagged reporter probe by incubating 80 µL of the HRP-streptavidin (10^−3^ g/mL) with 200 µL of the biotin-reporter probe sequence (10 µM) at room temperature for 45 min. The HRP-reporter probe conjugate was then stored at 4 °C until use. The conjugate pads (Conjugate Pad Grade 8975, Life Science-PALL Corp.) were cut to 16 × 10 mm. Prior to the deposition of the HRP-reporter probe conjugate, the conjugate pad was pretreated by immersion in 0.05 M Na_2_HPO_4_, pH 7.4 containing 0.1% Triton X-100, 0.5% bovine serum albumin, and 0.5% polyvinyl alcohol (MW 8,000) and dried overnight at 37 °C. Following pretreatment, the pads were stored at room temperature in a desiccator until conjugate application. The HRP-reporter probe conjugate was then applied to the conjugate pads by dipping the pads into the HRP-reporter probe conjugate solution and allowing it to migrate upwards. The conjugate pads were finally dried at room temperature in a desiccator where it was stored until use. 

#### 2.2.3. Nitrocellulose Membrane

For both the test line (capture probe) and control line (complement to the reporter probe), 10 µL of the biotin-labeled nucleic acid (3 × 10^−4^ M) was mixed with 10 µL streptavidin (10^−4^ M) and 30 µL of 0.04 M NaHCO_3_/Na_2_CO_3_ buffer, pH 9. The mixture was incubated for 20 min at room temperature with agitation to allow for conjugation of the biotin and streptavidin. The mixture was then deposited onto the nitrocellulose membrane (AE 98 FAST, Whatman^®^) (2 µL/cm) with an automated dispensing machine (Linomat IV, CAMAG, Wilmington, NC, USA). The membranes were placed in a vacuum oven at 40 °C for 1.5 h to dry, followed by blocking by immersing the strip (64 cm × 75 cm) in a blocking solution (0.015% casein sodium salt, 0.3% polyvinylpyrrolidone (MW 8,000), 0.001% Tween 20, in TBS, pH 8.6) for 1 min. The strip was then dried under vacuum at 25 °C for 2 h. Once dry, the membrane was stored in a desiccator until use.

#### 2.2.4. Absorbent Pad

The absorbent pad (CF5; Whatman International, Kent, England) was used as supplied without further modification. This pad serves as the capillary force driver as well as the reservoir for the used assay reagents.

### 2.3. Lateral Flow Assay Assembly

For assembly of the lateral flow assay, the sample pad, conjugate pad, nitrocellulose strip and absorbent pad were all placed in an overlapping sequence on a double sided adhesive tape as seen in Figure 1. The other side of the tape was mounted an acetate strip for rigidity. All components were cut to a width of 4 mm. The sample pad, conjugate pad, nitrocellulose and absorbent pads were cut to lengths of 10 mm, 10 mm, 25 mm, and 38 mm, respectively. The pads overlapped by 1 mm when assembled on the adhesive backing to allow for optimal flow.

### 2.4. Assay Procedure

**Figure 2:**
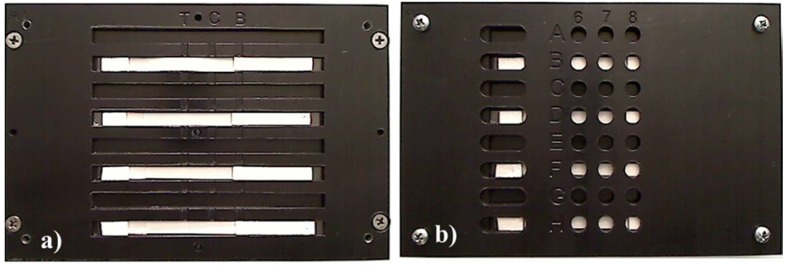
A holder device was machined in plastic in order for the test strips to be inserted into a plate reader. (**a**) The test strips were placed into the holder and then (**b**) the cover was placed over the test strips. The entire device had the same measurements as a 96-well plate, and the test and control lines corresponded to wells to allow for reading by a microtiter plate reader.

The assay ran as follows: 100 μL of sample solution were first applied onto the sample pad and allowed to migrate towards the absorbent pad. Ten minutes after the sample solution was applied, 100 μL of the 1:1 mixture of Super SigerSignal West Pico Luminol/Enhancer Solution and Supersignal West Pico Stable Peroxide Solution and H_2_O_2_ (Peirce Biotechnology, Rockford, IL, USA) were applied onto the sample pad. The intensities of chemiluminescent signals generated on the test and control lines were either quantified in a mitrotiter plate reader (Biotek Winooski, VT, USA) or it was imaged with a CCD imaging station (Kodak, Rochester, NY, USA). In order for the test strips to be read on a microtiter plate reader a device was fabricated to fit into a tray of the reader. The holder device was machined in Delrin™ using a CO_2_ laser (Epilog Laser, Golden, CO) and designed so that the test lines and control lines corresponded to a well location (Figure 2).

Up to eight test strips could be read at one time using the holder device. For the microtiter plate reader, the chemiluminescent intensity was quantified over time. The chemiluminescent/time curve was then integrated. CCD imaging was used for assay optimization and to generate images. Data are reported as averages ± standard deviation of at least n = 3 replicates. Results are representative of at least three independent experimental trials.

### 2.5. Assay Optimization

The concentrations of reagents (HRP and luminol/H_2_O_2_) were optimized both on a microtiter plate as well as on the strip in order to optimize signal to noise ratio. For the microtiter assay, 90 µL of the mixture of luminol and H_2_O_2_ (1/1, v/v) were added to a well of the clear 96-well microtiter plate. Then 10 µL of HRP-streptavidin solution with varying concentrations was added to the above wells in duplicate. The intensities of the chemiluminescent signal generated were detected by a CCD imager and quantified using ImageJ image analysis program (NIH).

Although the initial HRP-streptavidin concentrations were screened on a microtiter plate (see supplemental information), two concentrations were examined on the lateral flow. The two concentrations of HRP tested on the lateral flow strip were 10^−4^ and 10^−3^ g/mL.

## 3. Results and Discussion

### 3.1. Chemiluminescence Optimization

The volume of the mixture of luminol and H_2_O_2_ applied significantly influences the rate of the chemiluminescent reaction, and further affects the performance of the test strip. When the volume is too low, insufficient luminol and H_2_O_2_ migrates to the test and control lines, resulting in false negative results. When the volume is too high, the nitrocellulose membrane “floods”, causing the luminol/H_2_O_2_ mixture to move up the sides of the holder device by capillary action, preventing proper migration up the lateral flow test strip. Optimization of the volume of the luminol-H_2_O_2_ mixture demonstrated that the optimal volume applied was 110 μL. The rate of application of the target sample and the luminol-H_2_O_2_ mixture was further controlled to maintain uniform flow and migration of each component up the test strip.

### 3.2. Limit of Detection of the Test Strip

**Figure 3:**
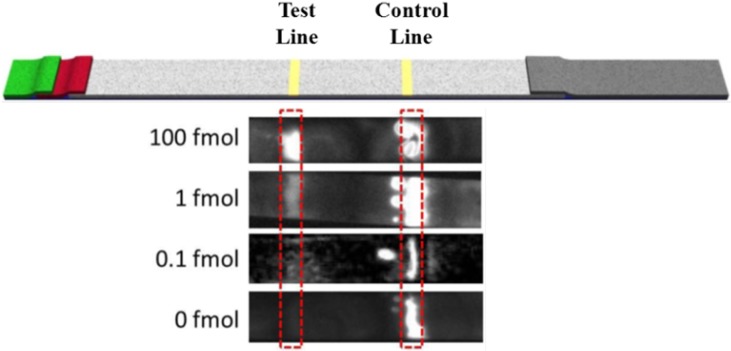
The CCD image of the lateral flow test strip tested in luminol-H_2_O_2_-HRP-chemiluminescent system taken by using a CCD imager. The image shows the low limit of detection with undetectable background signal using a luminol-based chemiluminescent system.

The effect of the target concentration on the chemiluminescent signal was visualized with a CCD camera (Figure 3). It can be seen that while the control line was present in all target levels tested, the signal generated at the test line decreased noticeably as the target levels decreased. This response was expected as the decrease in target would result in a decrease in HRP on the test line. 

**Figure 4:**
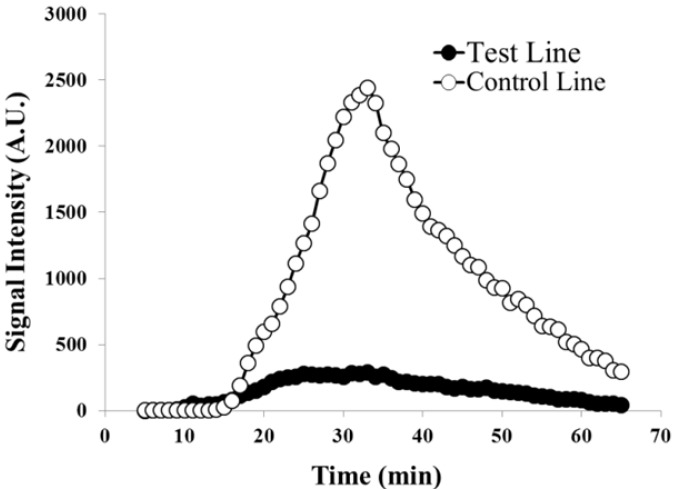
Chemiluminescence at both the test lines and controls lines were quantified using a microtiter plate reader over 60 min. At low target amounts such as this (0.1 fmol), the control line appears much stronger than the test line.

Similar results were obtained using the microtiter plate reader. This assay allowed for quantification of the signal over time. A typical result obtained using the multi-mode microtiter plate reader is shown in Figure 4. At low target amounts, the chemiluminescent signal generated at the control line was significantly stronger than that at the test line which is in agreement with the results from the CCD imaging. The chemiluminescence generated on the membrane was detectable for over an hour following the application of the luminol-H_2_O_2_ mixture. The results suggested an increase in the signal generation with increasing target levels. Generally, the maximum chemiluminescent signal intensity of the target samples occurred at 20–30 min after application of the mixture, and the maximum intensity of negative control was less than 40 min. To quantify the signal, the signal intensity was integrated over a sixty minute period. The integration of the test line signal increased accordingly with increasing target concentration.

**Figure 5:**
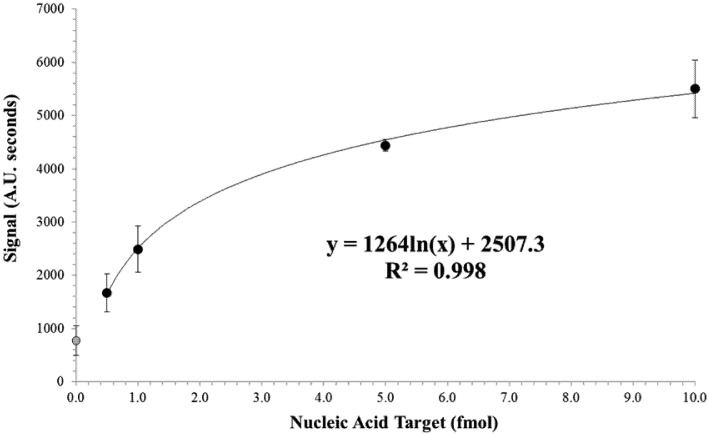
Dose response for increasing nucleic acid target. Signal area was calculated by integrating the test lines from the microtiter plate reader (Figure 4). The logarithmic model was fitted to the non-zero values tested. Data points represent an average of a minimum of three replicates and error bars represent the standard deviation. The grey data point at target concentration of zero represents the background signal and was not used in the fitting of the logarithmic model.

A logarithmic model was fitted to the dose response curve as seen in Figure 5 (R^2^ = 0.998). The limit of detection was determined using the 0 + 3SD method [28,29,30]. In this case, the background signal (*i.e.*, signal produced when the concentration of target analyte is zero) plus 3 times the SD at background are used as the minimum detectable sample. The corresponding target level was then determined using the logarithmic model. A limit of detection of approximately 0.5 fmol was determined by this 0 + 3SD method. A general linear model ANOVA was performed on the experimental data obtained at both background (0.0 fmol target analyte) and limit of detection (0.5 fmol target analyte) dose response data and statistical significance was determined at *P* < 0.01.

## 4. Conclusions

In this study, using *Trypanosoma* mRNA sequences as a model target, a nucleic acid and HRP-labeled lateral flow test strip chemiluminescent assay has been developed. The total assay time from sample application to results was approximately 70 min, but this time could be shortened at the sacrifice of limit of detection. The limit of detection was determined to be 0.5 fmol of target nucleic acid. Although synthetic targets are not expected to produce identical results as would target mRNA, the results still compare favorably to assays using similar synthetic targets. The results suggest that a general nucleic acid and enzyme-labeled lateral flow test strip chemiluminescent assay can be developed to rapidly, sensitively, and specifically test various pathogens and diseases. The reported assay can be adapted for detection of a range of pathogens or other analytes, and can be modified to detect RNA, DNA, antibodies, *etc*. Our novel membrane-to-microplate reader adapter was an effective and convenient means to quantify several lateral flow assays simultaneously, enabling multiplexed or replicate analysis in parallel. The assay is expected to be used for the rapid, sensitive and specific mass screening of samples, and the rapid and accurate detection of the pathogens and diseases so that the prevention, diagnosis and treatments of the diseases is greatly improved. Future work will demonstrate this with isolated *Trypanosoma* mRNA from clinical samples. 

## Supplementary Files

Supplementary File 1 PDF-Document (PDF, 249 KB)
