# Supplementary material for: Development of Chemiluminescent Lateral Flow Assay for the Detection of Nucleic Acids
Source: Biosensors (Basel). 2012 Jan 18;2(1):32–42. doi: 10.3390/bios2010032 (PMC4263543; doi:10.3390/bios2010032)
Supplement: Supplementary File 1 — PDF-Document (PDF, 249 KB) [file biosensors-02-00032-s001.pdf]

Supplementary Information

Yuhong Wang, Catherine Fill and Sam R. Nugen *

Department of Food Science, University of Massachusetts, 102 Holdsworth Way, Amherst, MA 01003, USA; E-Mails: yuhongw@foodsci.umass.edu (Y.W.); cfill@foodsci.umass.edu (C.F.)

* Author to whom correspondence should be addressed; E-Mail: snugen@foodsci.umass.edu; Tel.: +001-413-545-1025; Fax: +001-413-545-1262.

Received: 8 December 2011; in revised form: 6 January 2012 / Accepted: 16 January 2012 / Published: 18 January 2012

For the microtiter assay, 90 µL of the mixture of luminol and H_2O_2 (1/1, v/v) was added to a well of the clear 96-well microtiter plate. Then 10 µL of HRP-streptavidin solution with varying concentrations (10^{-7}, 10^{-6}, 10^{-5}, 10^{-4}, and 10^{-3} g/mL) was added to the above wells in duplicate. In addition, 90 µL of the mixture of luminol and H_2O_2 (1/1, v/v) was also used as a negative control. The intensities of the chemiluminescent signal generated were detected by a CCD imager and quantified using ImageJ image analysis program (NIH). The CCD images shown that intensities of the chemiluminescent signal generated for the concentrations of 10^{-7} and 10^{-6} g/mL were very weak and close to the results of the negative control; The signal intensities for the concentrations of 10^{-4} and 10^{-3} g/mL were very strong; The signal intensity for the concentrations of 10^{-5} g/mL was obviously stronger and weaker than those of negative control, and the concentrations of 10^{-4} and 10^{-3} g/mL, respectively (see the Figure S1 below).

Figure S1. Screening of initial HRP-streptavidin concentrations.

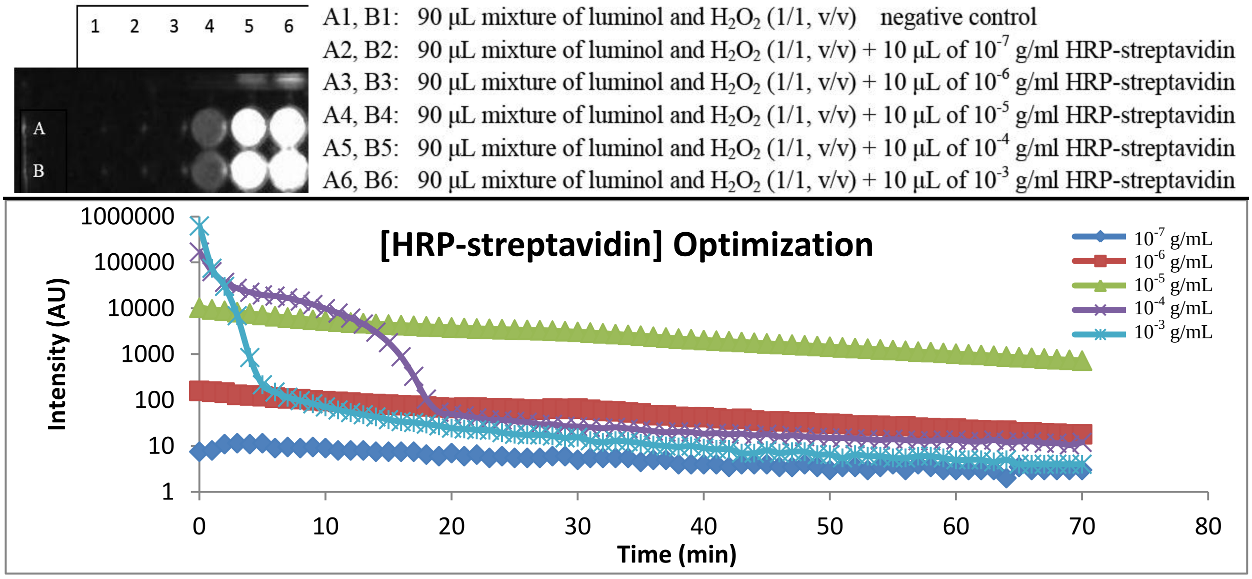

The intensities of chemiluminescence signals were also quantified by using a multi-mode microplate reader (Synergy^{TM} 2, BioTek Instruments, Inc., Winooski, VM, USA). Firstly, 90 µL of the mixture of luminol and H_2O_2 (1/1, v/v) was added to a well of the black 96-well microtiter plate. Then 10 µL of HRP-streptavidin solution with varying concentrations (10^{-7}, 10^{-6}, 10^{-5}, 10^{-4}, and 10^{-3} g/mL) was added to the above wells in duplicate. In addition, 90 µL of the mixture of luminol and H_2O_2 (1/1, v/v) was also used as a negative control. Finally, the intensities of the chemiluminescent signals generated were continuously detected and recorded with 1 min of time interval. The signal intensities as a function of time at different HRP-streptavidin concentrations were shown in Figure S1, which demonstrated the similar results to the CCD imager testing. Therefore 10^{-4} and 10^{-3} g/mL were chosen as two quantity levels of HRP-streptavidin to prepare the conjugate pad in order to obtain low limits of detection. Although the initial HRP-streptavidin concentrations were screened on a microtiter plate, two concentrations (10^{-4} and 10^{-3} g/mL) were examined on the lateral flow. Experimental results from the lateral flow assay demonstrated that chemiluminescence signals at HRP-streptavidin concentrations of 10^{-4} and 10^{-3} g/mL were strong (we also did experiments for the concentration of 10^{-5} g/mL, but the signals were very weak, so we did not report). Therefore, two concentrations of HRP-streptavidin conjugate were used to prepare the conjugate pad (10^{-4} and 10^{-3} g/mL). The limits of detection were determined **under the fixed concentration of HRP-streptavidin**.

© 2012 by the authors; licensee MDPI, Basel, Switzerland. This article is an open access article distributed under the terms and conditions of the Creative Commons Attribution license (http://creativecommons.org/licenses/by/3.0/).
